# Supplementary material for: MITOL deficiency triggers hematopoietic stem cell apoptosis via ER stress response
Source: EMBO J. 2024 Jan 18;43(3):2. doi: 10.1038/s44318-024-00029-0 (PMC10897143; doi:10.1038/s44318-024-00029-0)
Supplement: Supplementary file 1 — Table EV1 [file 44318_2024_29_MOESM1_ESM.docx]

**Table EV1**

Mouse primers sequence information for quantitative PCR is listed as below. 5’→3’

| *Bax* | Forward | CCGGCGAATTGGAGATGAACT |
| --- | --- | --- |
|  | Reverse | CCAGCCCATGATGGTTCTGAT |
| *Bak1* | Forward | GGAATGCCTACGAACTCTTCACC |
|  | Reverse | CAAACCACGCTGGTAGACGTAC |
| *Rps18* | Forward | CGGAAAATAGCCTTCGCCATCAC |
|  | Reverse | ATCACTCGCTCCACCTCATCCT |
| *Marchf5* | Forward | CCGCCTCTCAGTGCTATTGTC |
|  | Reverse | GCAAAGCACACCCAGCAAC |
| *Gapdh* | Forward | GACTTCAACAGCAACTCCCAC |
|  | Reverse | TCCACCACCCTGTTGCTGTA |
