# Supplementary material for: MITOL deficiency triggers hematopoietic stem cell apoptosis via ER stress response
Source: EMBO J. 2024 Jan 18;43(3):2. doi: 10.1038/s44318-024-00029-0 (PMC10897143; doi:10.1038/s44318-024-00029-0)
Supplement: Supplementary file 2 — Table EV2 [file 44318_2024_29_MOESM2_ESM.docx]

**Table EV2**

Software and algorithms.

| Software | Version | Source |
| --- | --- | --- |
| STAR | 2.7.10a | https://anaconda.org/bioconda/star |
| samtools | 1.10 | https://anaconda.org/bioconda/samtools |
| subread | 2.0.0 | https://anaconda.org/bioconda/subread |
| RStudio | 2022.07.1+554 | https://www.rstudio.com/ |
| R | 4.1.2 | <https://www.r-project.org/> |
| pheatmap | 1.0.12 | https://cran.r-project.org/web/packages/pheatmap/index.html |
| ggplot2 | 3.3.6 | https://cran.r-project.org/web/packages/ggplot2/index.html |
| DEseq2 | 1.36.0 | https://bioconductor.org/packages/release/bioc/html/DESeq2.html |
| EnhancedVolcano | 1.14.0 | https://bioconductor.org/packages/release/bioc/html/EnhancedVolcano.html |
| Enrichr | 3.1.0 | https://maayanlab.cloud/Enrichr/ |
