# Supplementary figures and images for: MITOL deficiency triggers hematopoietic stem cell apoptosis via ER stress response
Source: EMBO J. 2024 Jan 18;43(3):2. doi: 10.1038/s44318-024-00029-0 (PMC10897143; doi:10.1038/s44318-024-00029-0)

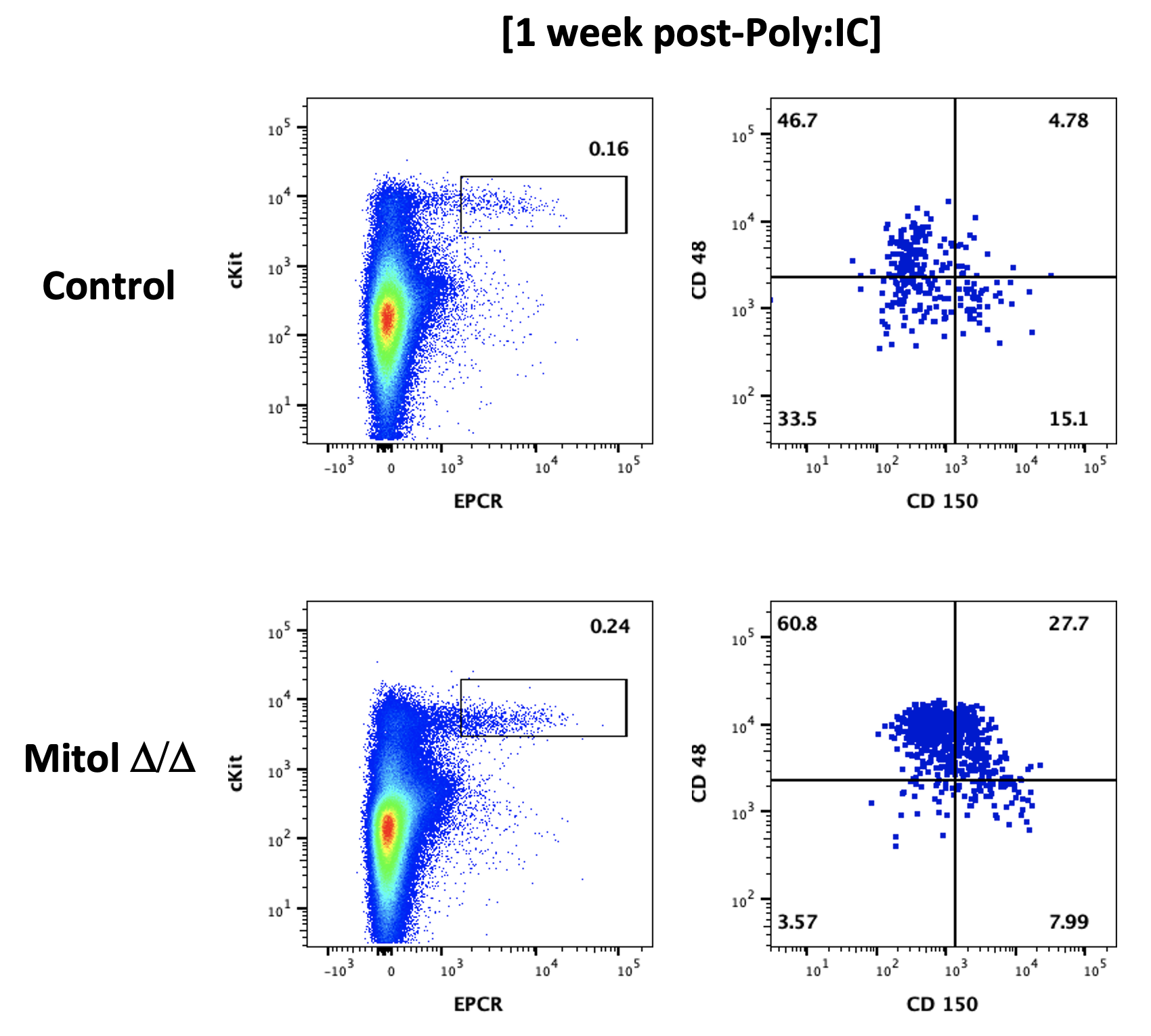

Supplement: Supplementary file 3 — Source Data Fig. 1 [file 44318_2024_29_MOESM3_ESM.zip › Figure 1/1E/1E_Flow cytometry.tiff]

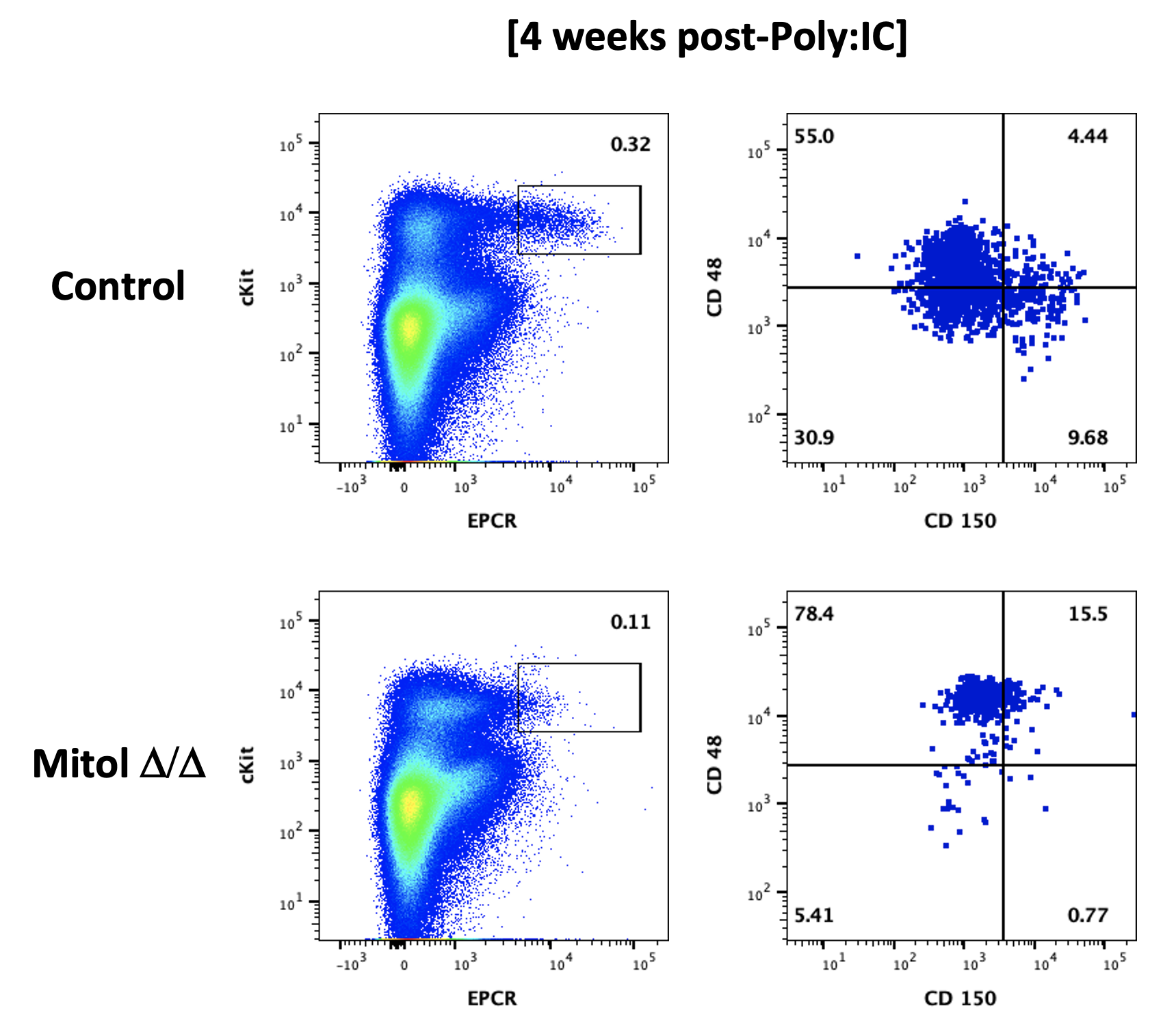

Supplement: Supplementary file 3 — Source Data Fig. 1 [file 44318_2024_29_MOESM3_ESM.zip › Figure 1/1C/1C_Flow cytometry.tiff]

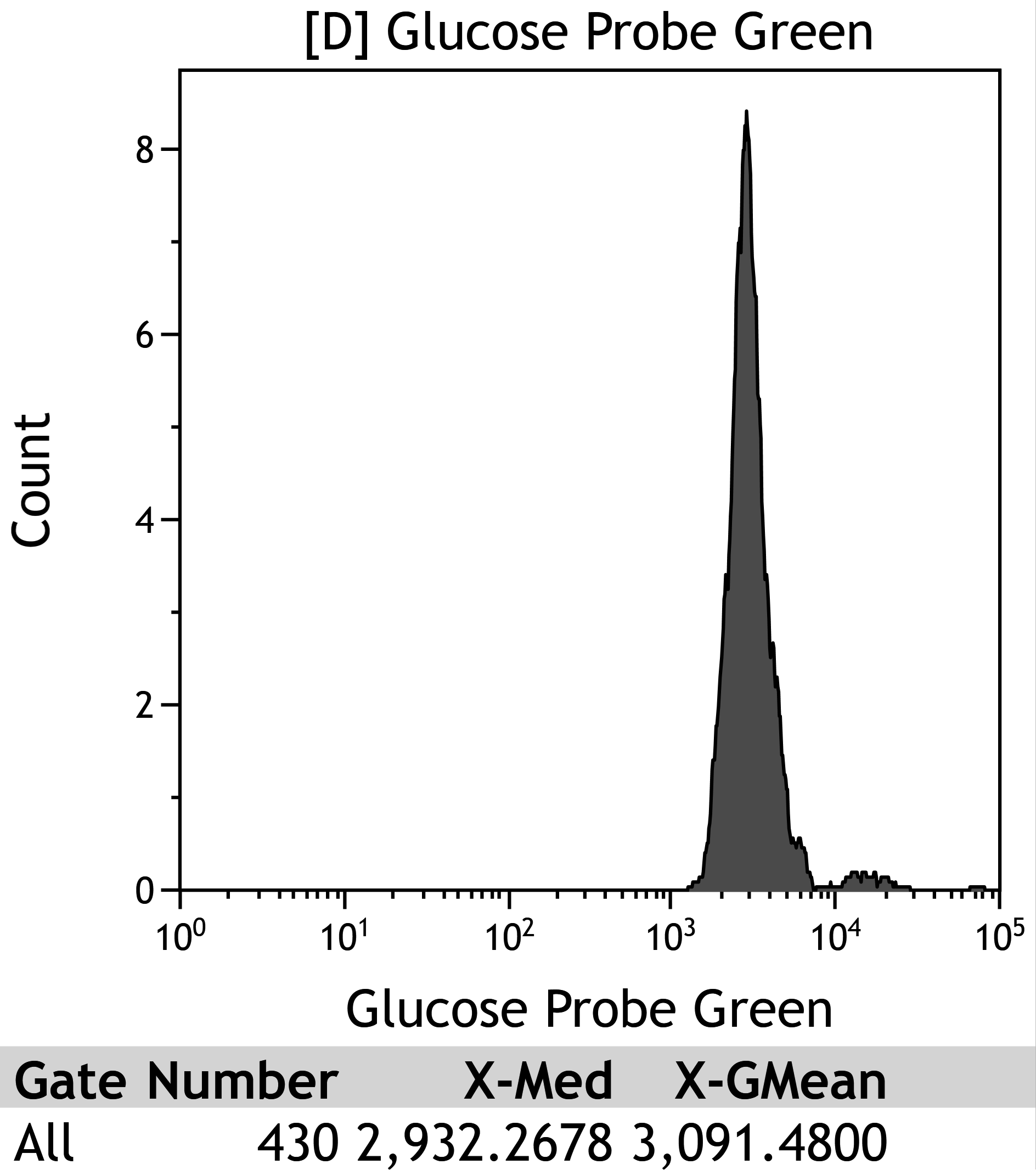

Supplement: Supplementary file 6 — Source Data Fig. 4 [file 44318_2024_29_MOESM6_ESM.zip › Figure 4/4E/Wt-Glucose.tiff]

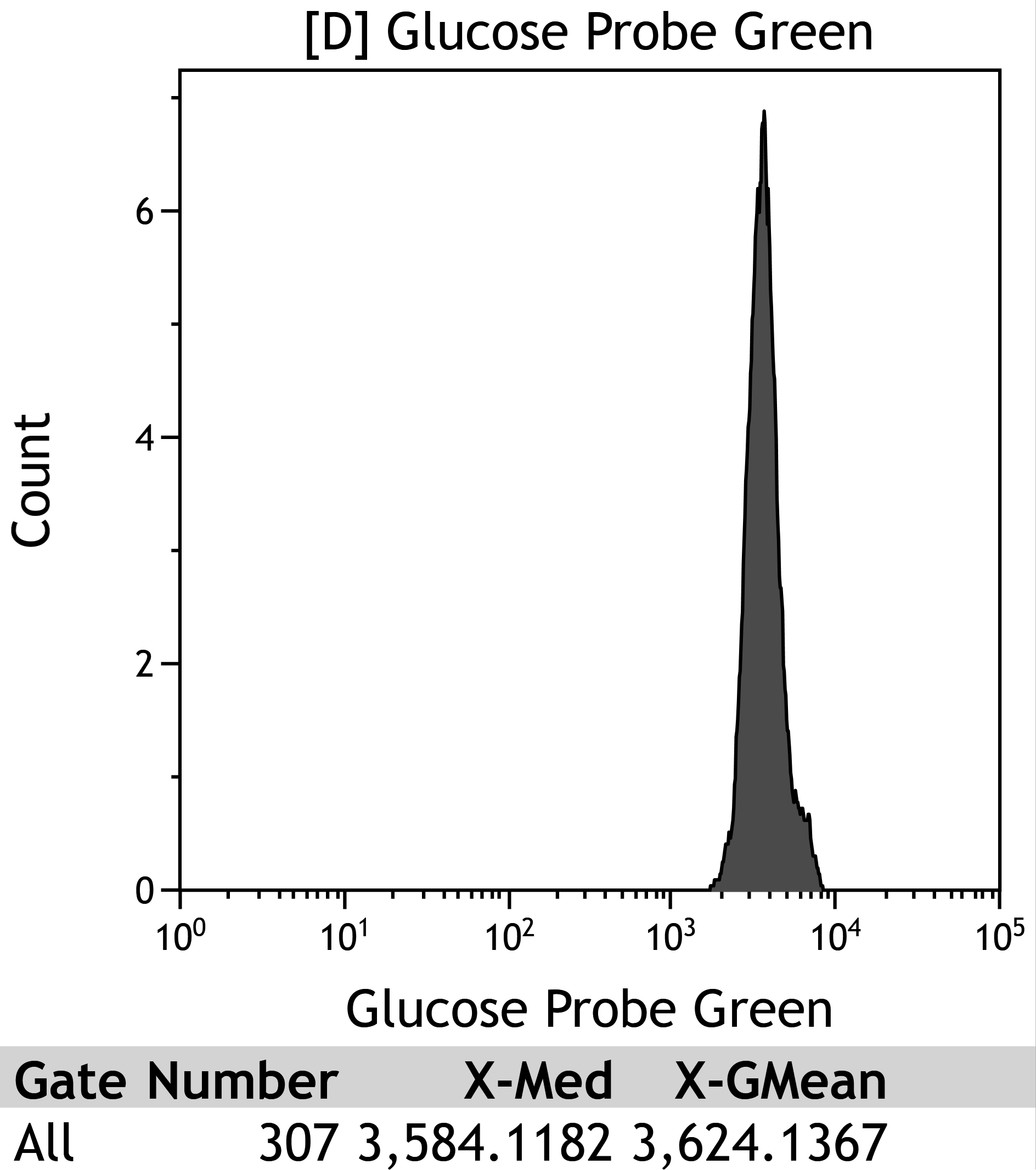

Supplement: Supplementary file 6 — Source Data Fig. 4 [file 44318_2024_29_MOESM6_ESM.zip › Figure 4/4E/Mitol KO-Glucose.tiff]

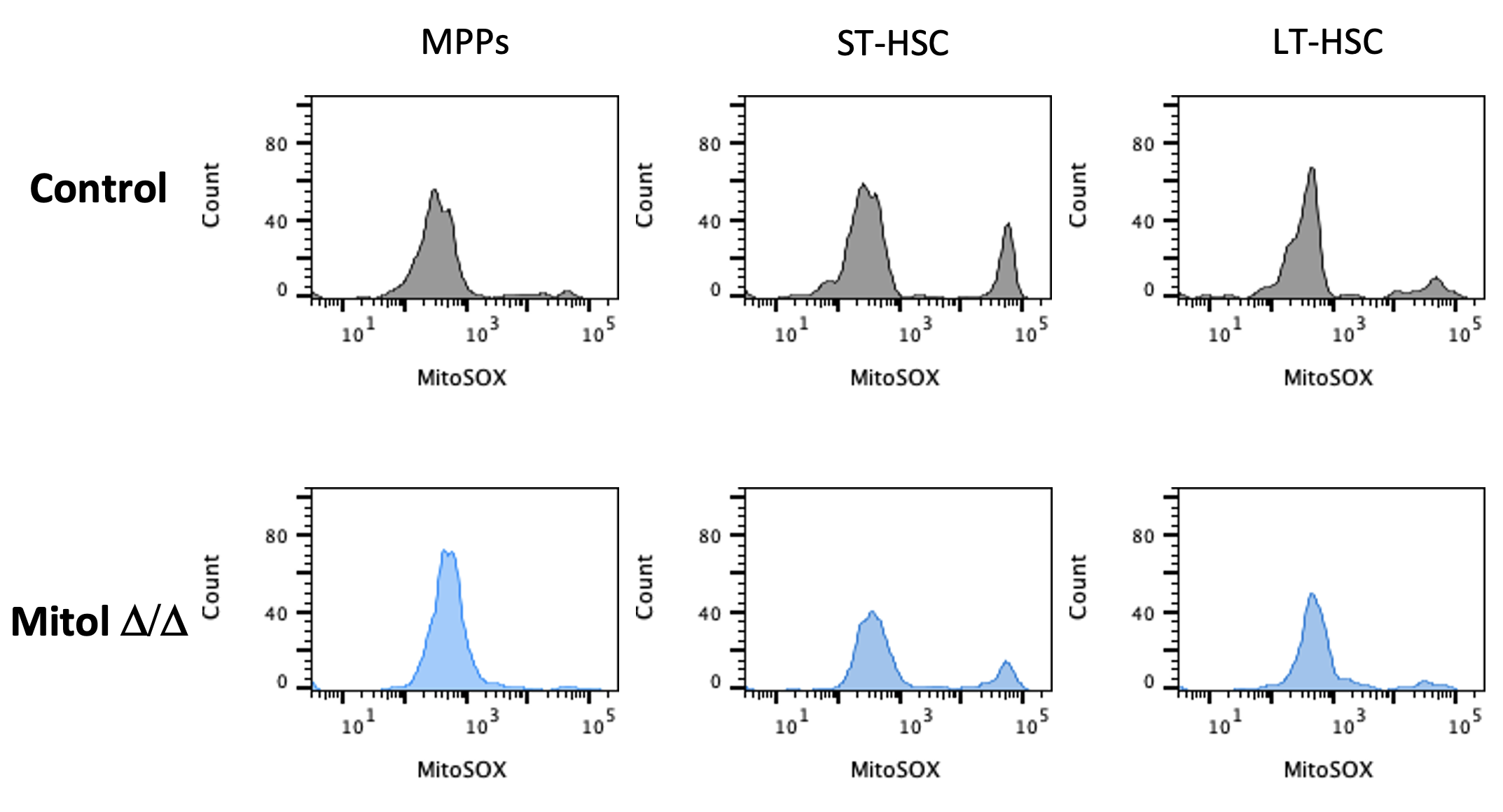

Supplement: Supplementary file 6 — Source Data Fig. 4 [file 44318_2024_29_MOESM6_ESM.zip › Figure 4/4C/4C_Flow cytometry.tiff]

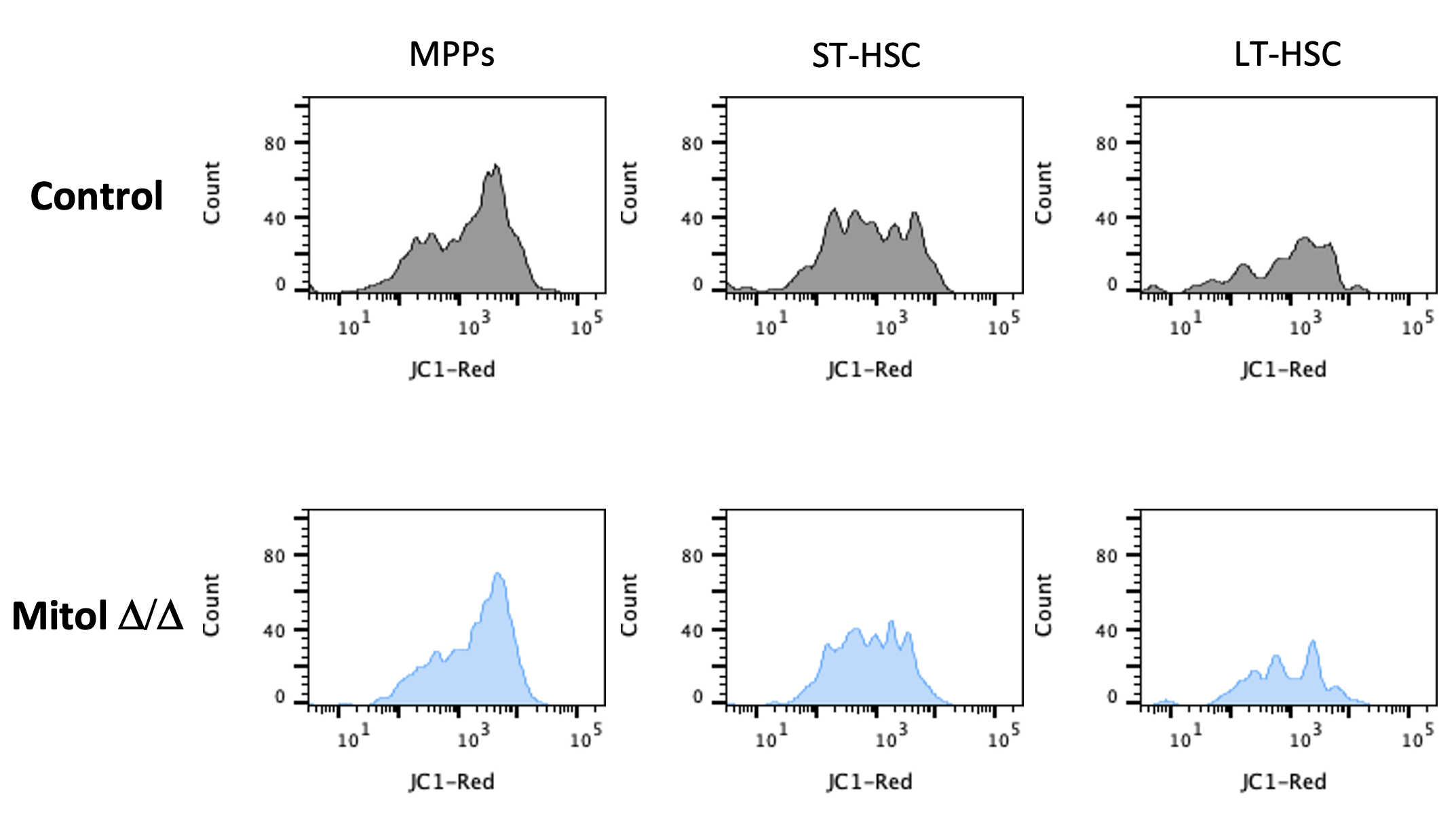

Supplement: Supplementary file 6 — Source Data Fig. 4 [file 44318_2024_29_MOESM6_ESM.zip › Figure 4/4A/4A_Flow cytometry.tiff]

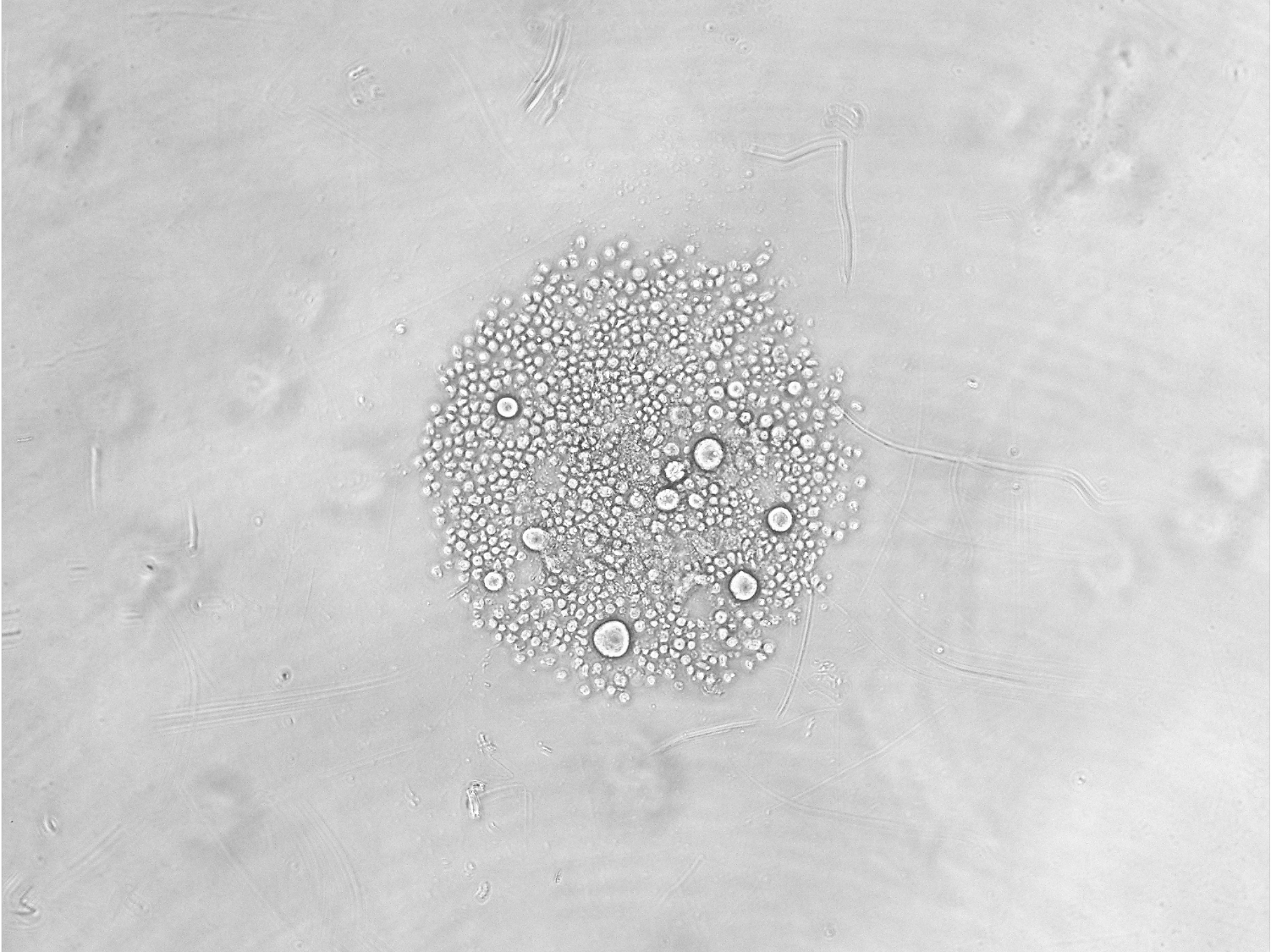

Supplement: Supplementary file 7 — Source Data Fig. 5 [file 44318_2024_29_MOESM7_ESM.zip › Figure 5/5A/Mitol KO_1w culture.tiff]

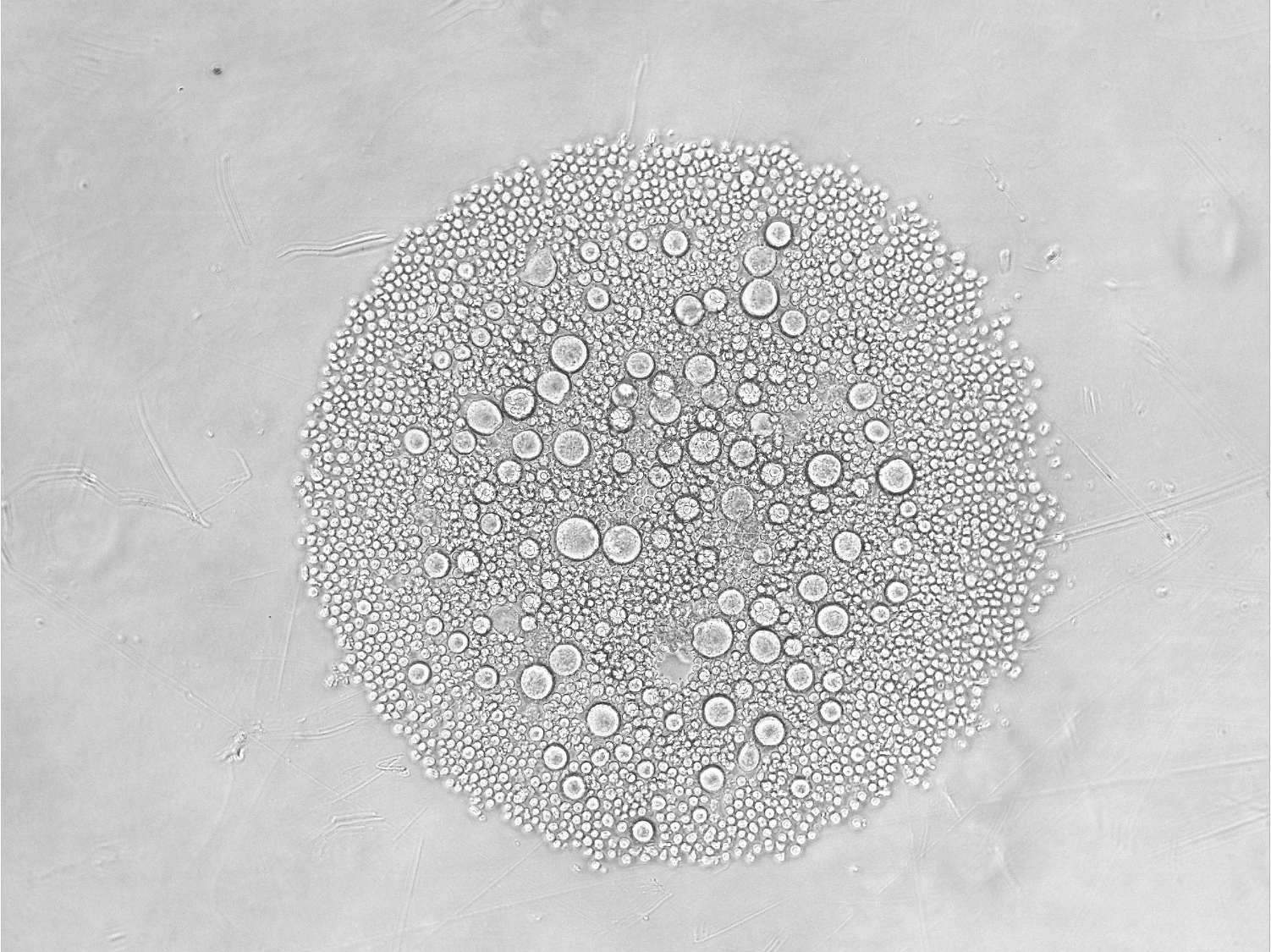

Supplement: Supplementary file 7 — Source Data Fig. 5 [file 44318_2024_29_MOESM7_ESM.zip › Figure 5/5A/Control_1w culture.tiff]

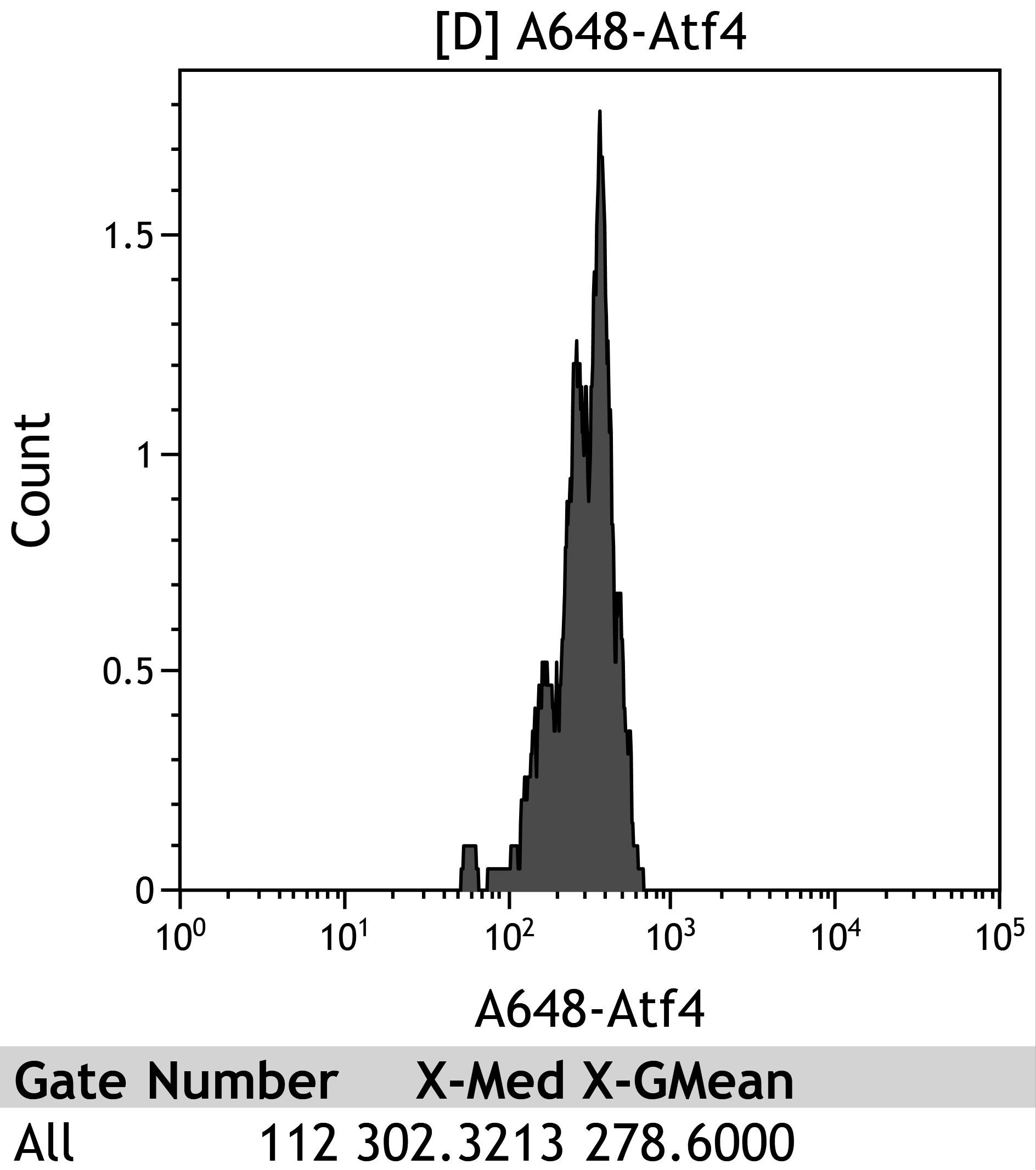

Supplement: Supplementary file 7 — Source Data Fig. 5 [file 44318_2024_29_MOESM7_ESM.zip › Figure 5/5F/Mitol KO-Atf4.tiff]

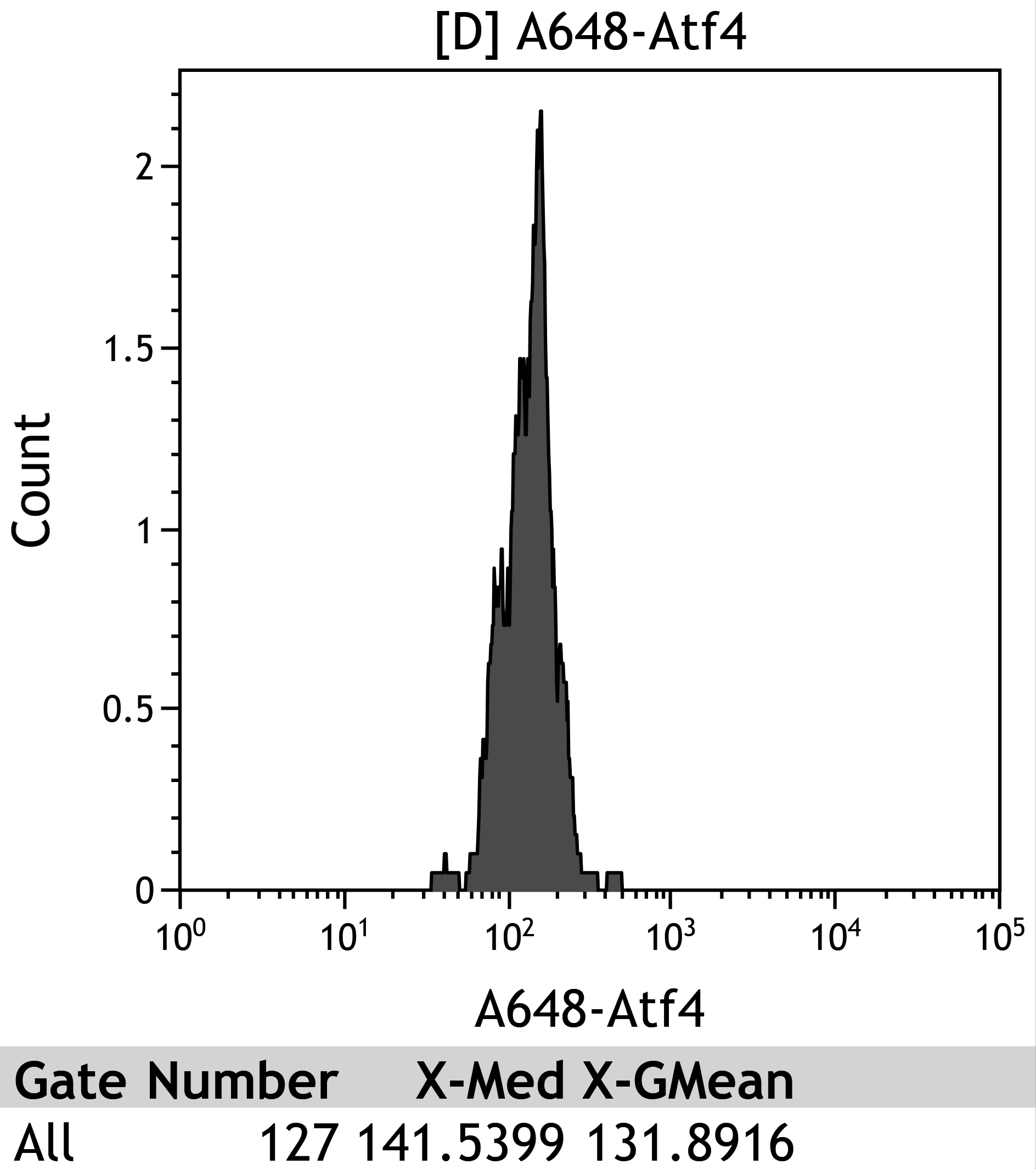

Supplement: Supplementary file 7 — Source Data Fig. 5 [file 44318_2024_29_MOESM7_ESM.zip › Figure 5/5F/Wt-Atf4.tiff]

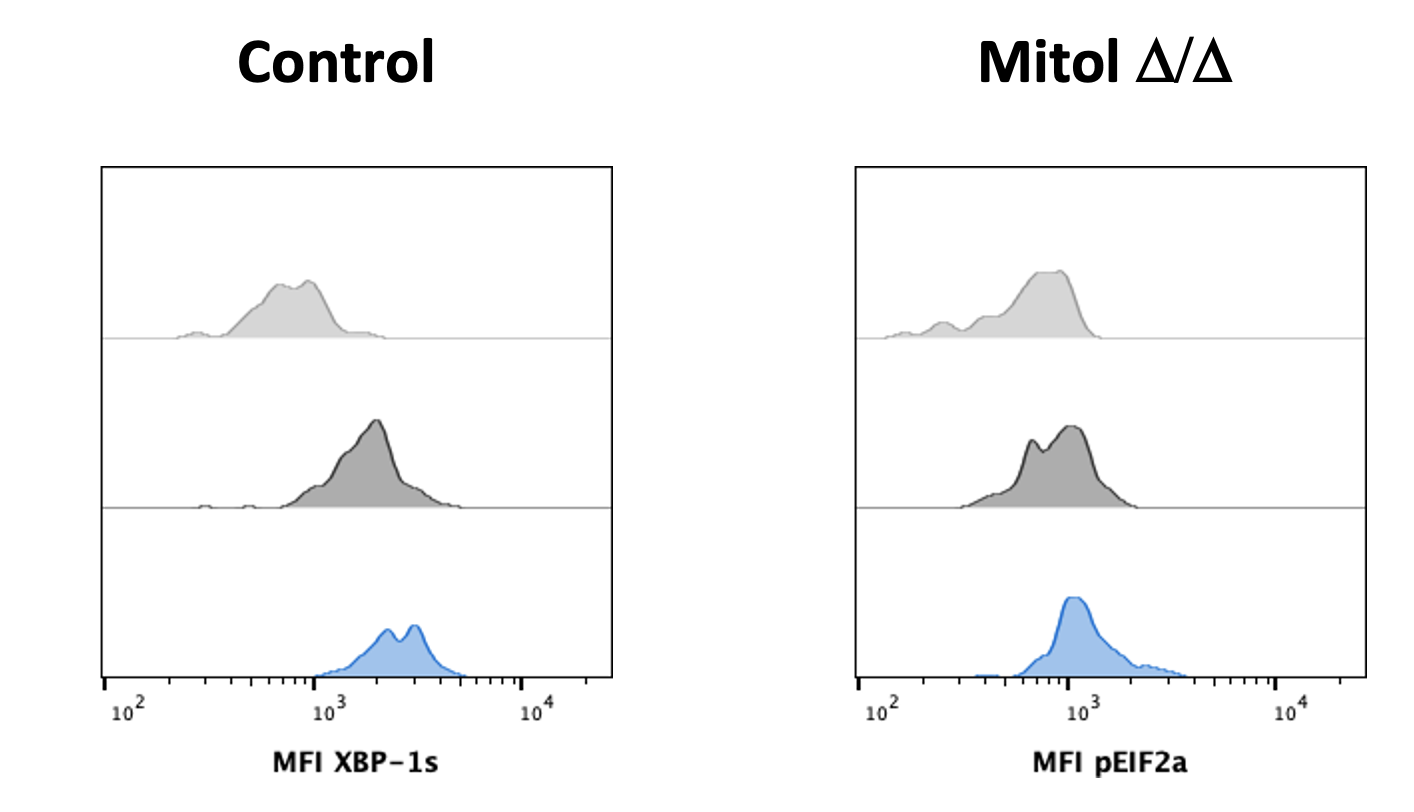

Supplement: Supplementary file 7 — Source Data Fig. 5 [file 44318_2024_29_MOESM7_ESM.zip › Figure 5/5D/5D_Flow cytometry.tiff]

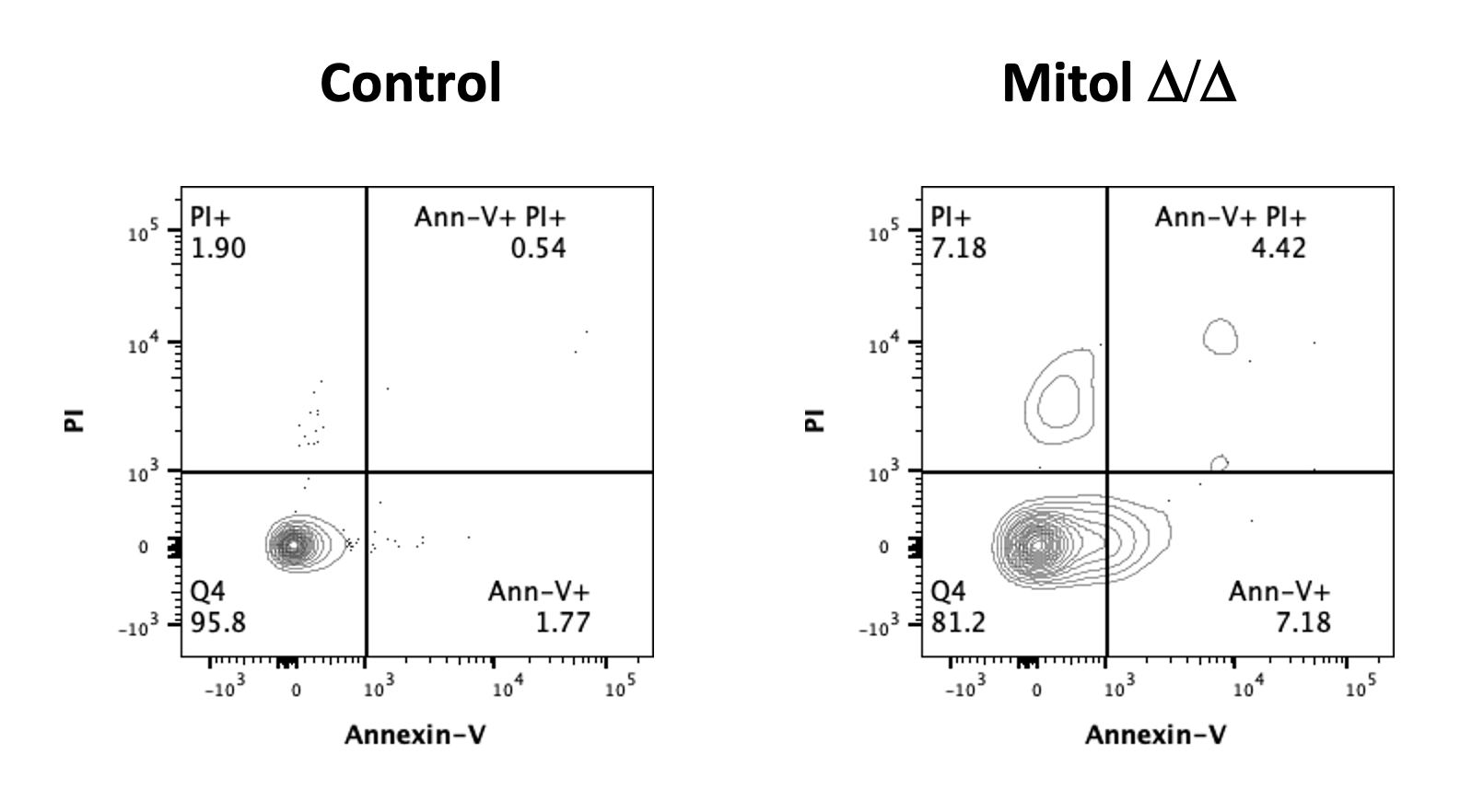

Supplement: Supplementary file 7 — Source Data Fig. 5 [file 44318_2024_29_MOESM7_ESM.zip › Figure 5/5B/5B_Flow cytometry.tiff]

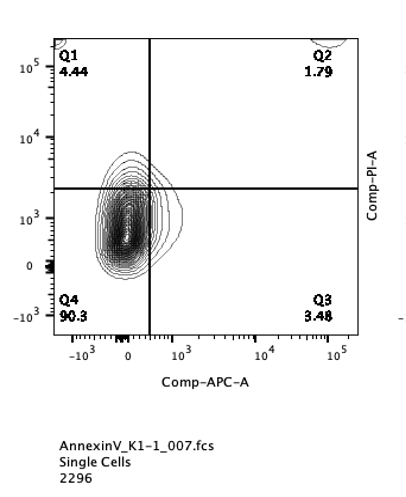

Supplement: Supplementary file 8 — Source Data Fig. 6 [file 44318_2024_29_MOESM8_ESM.zip › Figure 6/6A/KO-control.tiff]

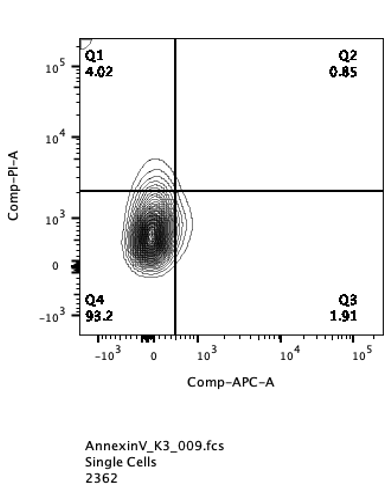

Supplement: Supplementary file 8 — Source Data Fig. 6 [file 44318_2024_29_MOESM8_ESM.zip › Figure 6/6A/KO-KIRA6-0.1uM.tiff]

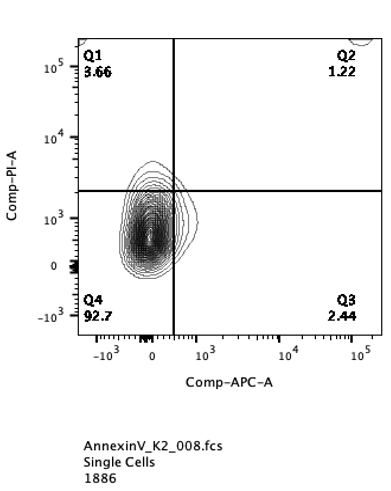

Supplement: Supplementary file 8 — Source Data Fig. 6 [file 44318_2024_29_MOESM8_ESM.zip › Figure 6/6A/KO-KIRA6-0.03uM.tiff]

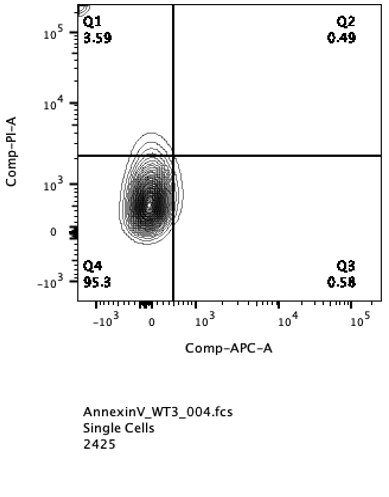

Supplement: Supplementary file 8 — Source Data Fig. 6 [file 44318_2024_29_MOESM8_ESM.zip › Figure 6/6A/WT-KIRA6-0.1uM.tiff]

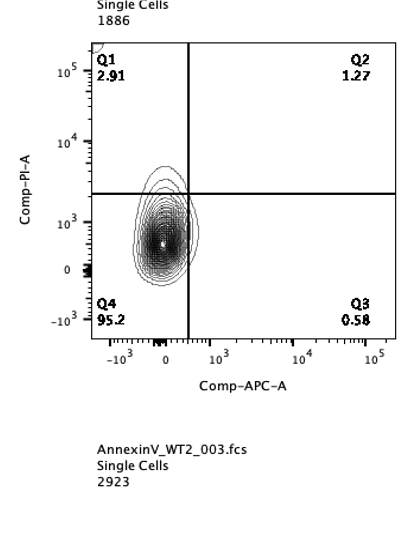

Supplement: Supplementary file 8 — Source Data Fig. 6 [file 44318_2024_29_MOESM8_ESM.zip › Figure 6/6A/WT-KIRA6-0.03uM.tiff]

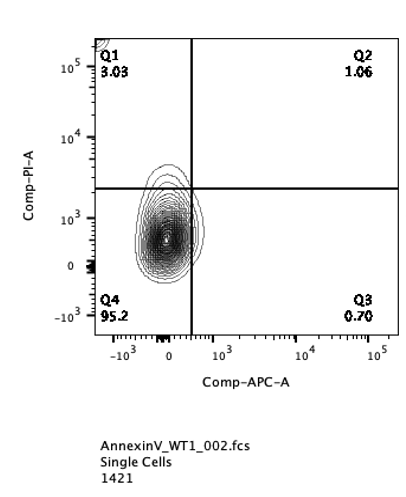

Supplement: Supplementary file 8 — Source Data Fig. 6 [file 44318_2024_29_MOESM8_ESM.zip › Figure 6/6A/WT-control.tiff]
