# Supplementary material for: MITOL deficiency triggers hematopoietic stem cell apoptosis via ER stress response
Source: EMBO J. 2024 Jan 18;43(3):2. doi: 10.1038/s44318-024-00029-0 (PMC10897143; doi:10.1038/s44318-024-00029-0)
Supplement: Supplementary file 10 — Expanded View Figures [file 44318_2024_29_MOESM10_ESM.pdf]

Expanded View Figures

Figure EV1. Features of Mitol$^{\Delta/\Delta}$ HSCs.

(**A**) Peripheral blood populations after 1 weeks of Poly:IC induced Mitol deletion ($n = 3$). (**B**) PCA analysis of RNA-seq generated from murine control and Mitol$^{\Delta/\Delta}$ HSCs after 1 week post Poly:IC induction. (**C**) Heatmap of top 100 DEGs from RNA-seq analysis. (**D**, **E**) Cell cycle analysis of HSPCs 1 week post Poly:IC induction shown by FACS (**D**) and cell percentage at different cell cycle stages shown in (**E**) ($n = 3$). Data Information: Data represent mean ± SEM with two-tailed unpaired Student's t test. ns, $P > 0.05$; $^*P < 0.05$; $^{**}P < 0.01$.

© The Author(s)

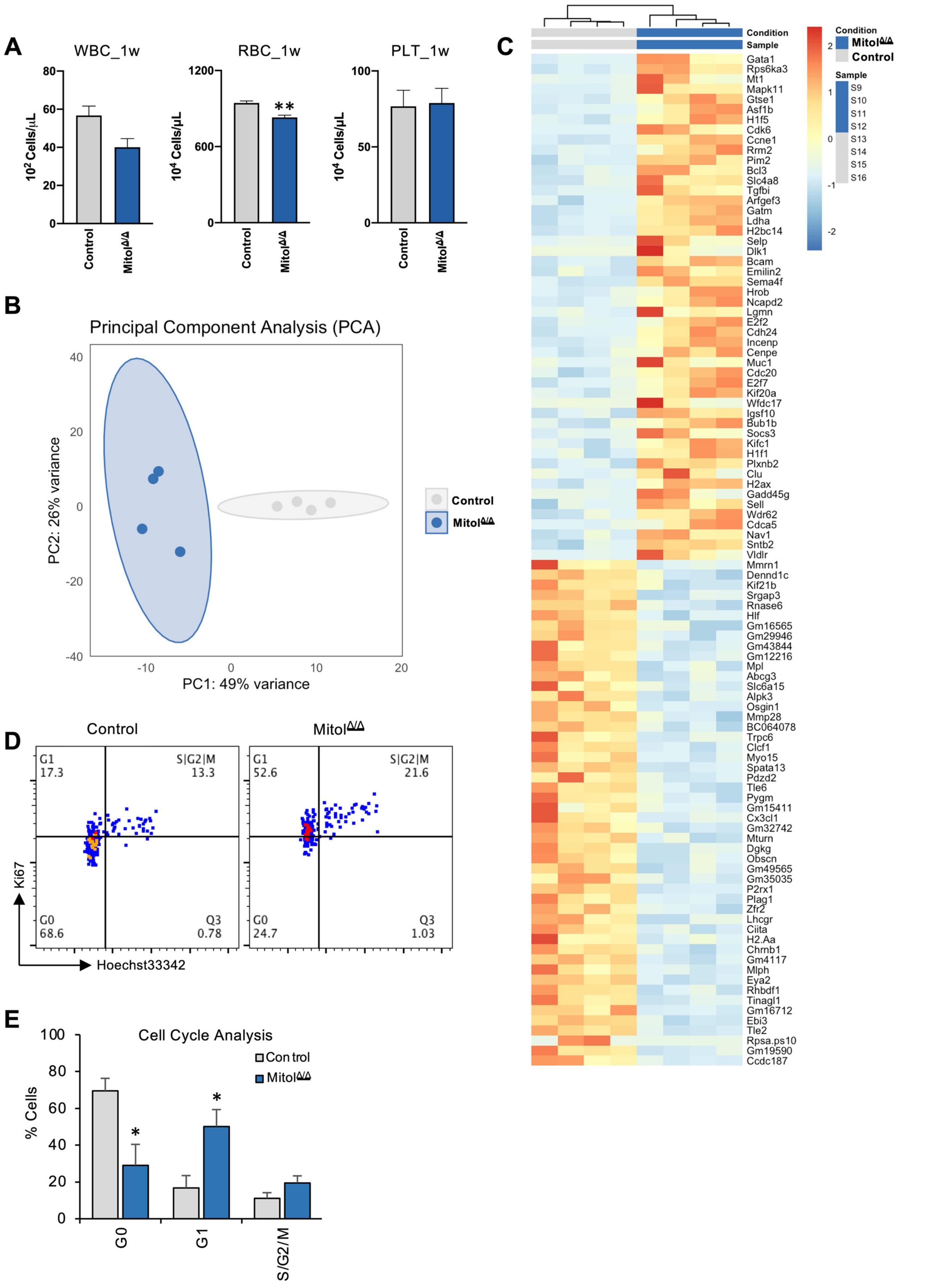
A
WBC_1w
RBC_1w
PLT_1w
10² Cells/µL
10⁴ Cells/µL
Control
MitolΔ/Δ
B
Principal Component Analysis (PCA)
PC2: 26% variance
PC1: 49% variance
Control
MitolΔ/Δ
C
Condition
Sample
S9
S10
S11
S12
S13
S14
S15
S16
D
Control
MitolΔ/Δ
G1 17.3
S|G2|M 13.3
G0 68.6
Q3 0.78
G1 52.6
S|G2|M 21.6
G0 24.7
Q3 1.03
Ki67
Hoechst33342
E
Cell Cycle Analysis
% Cells
G0
G1
S/G2/M

 © The Author(s)

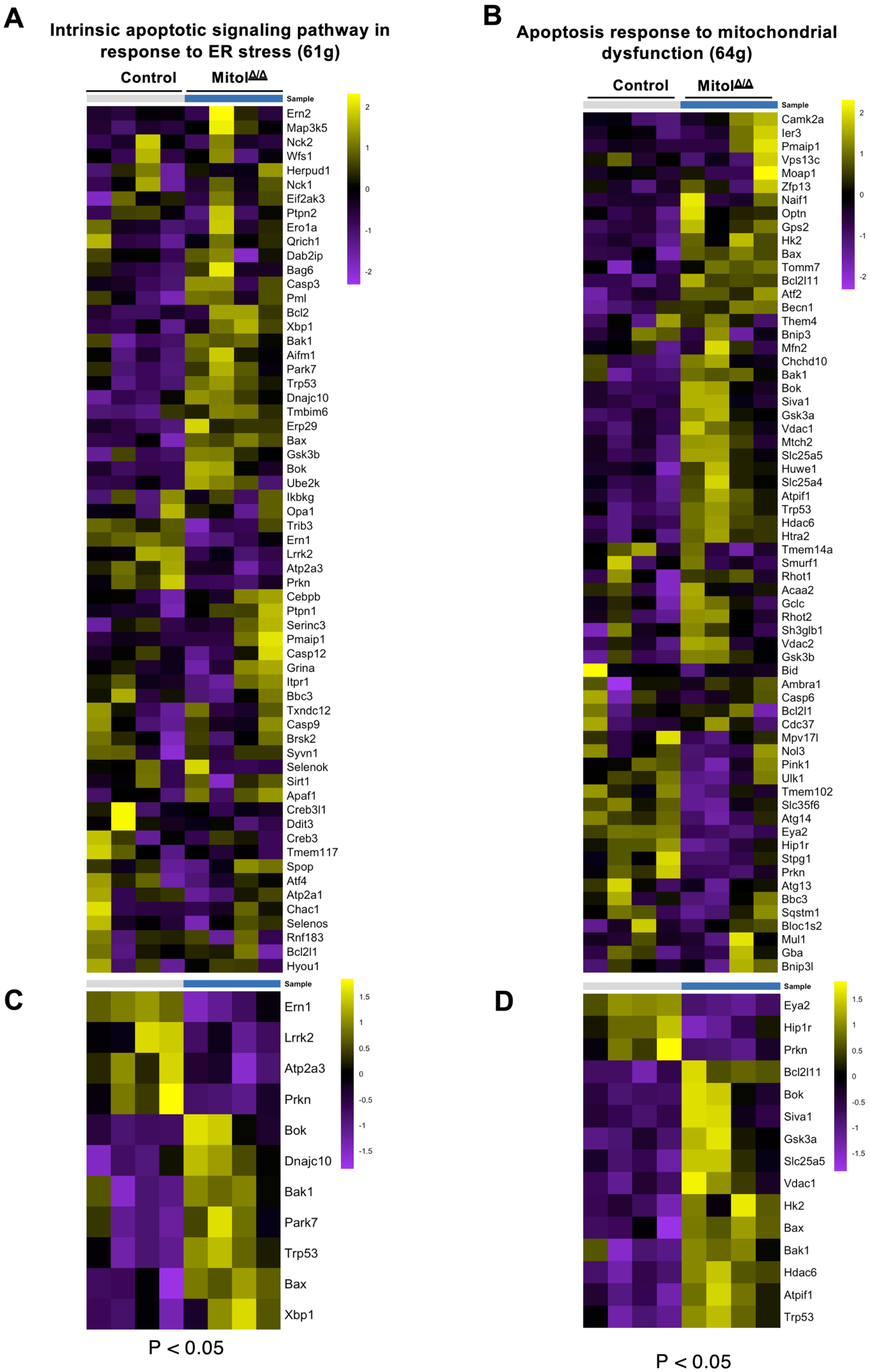
A
Intrinsic apoptotic signaling pathway in response to ER stress (61g)
Control
MitolΔ/Δ
Sample
2
1
0
-1
-2
Ern2
Map3k5
Nck2
Wfs1
Herpud1
Nck1
Eif2ak3
Ptpn2
Ero1a
Qrich1
Dab2ip
Bag6
Casp3
Pml
Bcl2
Xbp1
Bak1
Aifm1
Park7
Trp53
Dnajc10
Tmbim6
Erp29
Bax
Gsk3b
Bok
Ube2k
Ikbkg
Opa1
Trib3
Ern1
Lrrk2
Atp2a3
Prkn
Cebpb
Ptpn1
Serinc3
Pmaip1
Casp12
Grina
Itpr1
Bbc3
Txndc12
Casp9
Brsk2
Syvn1
Selenok
Sirt1
Apaf1
Creb3l1
Ddit3
Creb3
Tmem117
Spop
Atf4
Atp2a1
Chac1
Selenos
Rnf183
Bcl2l1
Hyou1
B
Apoptosis response to mitochondrial dysfunction (64g)
Control
MitolΔ/Δ
Sample
2
1
0
-1
-2
Camk2a
Ier3
Pmaip1
Vps13c
Moap1
Zfp13
Naif1
Optn
Gps2
Hk2
Bax
Tomm7
Bcl2l11
Atf2
Becn1
Them4
Bnip3
Mfn2
Chchd10
Bak1
Bok
Siva1
Gsk3a
Vdac1
Mtch2
Slc25a5
Huwe1
Slc25a4
Atpif1
Trp53
Hdac6
Htra2
Tmem14a
Smurf1
Rhot1
Acaa2
Gclc
Rhot2
Sh3glb1
Vdac2
Gsk3b
Bid
Ambra1
Casp6
Bcl2l1
Cdc37
Mpv17l
Nol3
Pink1
Ulk1
Tmem102
Slc35f6
Atg14
Eya2
Hip1r
Stpg1
Prkn
Atg13
Bbc3
Sqstm1
Bloc1s2
Mul1
Gba
Bnip3l
C
Sample
1.5
1
0.5
0
-0.5
-1
-1.5
Ern1
Lrrk2
Atp2a3
Prkn
Bok
Dnajc10
Bak1
Park7
Trp53
Bax
Xbp1
P < 0.05
D
Sample
1.5
1
0.5
0
-0.5
-1
-1.5
Eya2
Hip1r
Prkn
Bcl2l11
Bok
Siva1
Gsk3a
Slc25a5
Vdac1
Hk2
Bax
Bak1
Hdac6
Atpif1
Trp53
P < 0.05

Figure EV2. Genes involved in ER and mitochondrial-regulated apoptosis.

(**A–D**) Heatmaps of Intrinsic apoptotic signaling pathway in response to ER stress (**A**) with significantly upregulated genes (**C**) and apoptosis response to mitochondrial dysfunction (**B**) with significantly upregulated genes (**D**). Data Information: Data was analyzed with the Wald test.

© The Author(s)

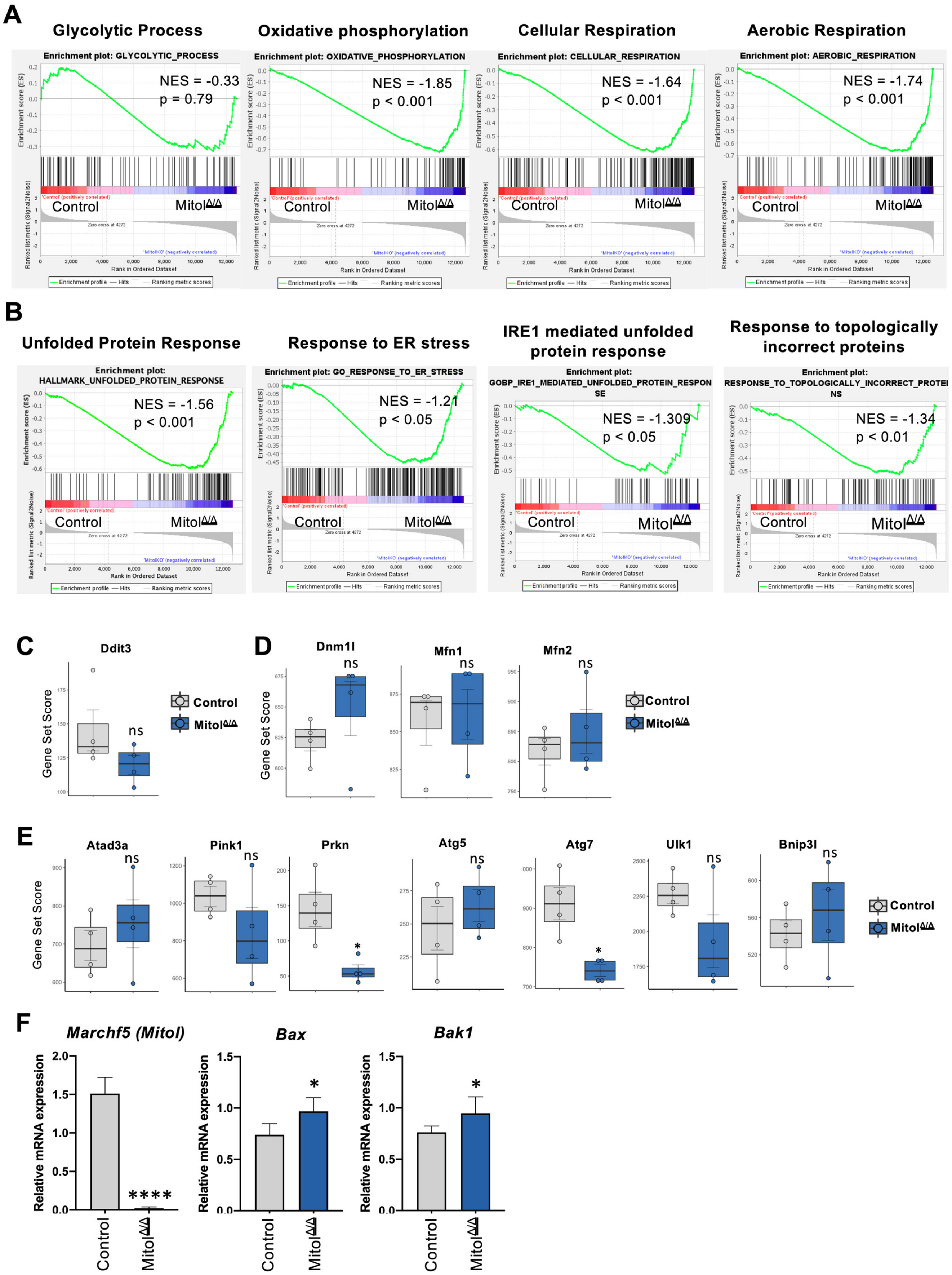
A
Glycolytic Process
Enrichment plot: GLYCOLYTIC_PROCESS
NES = -0.33
p = 0.79
Oxidative phosphorylation
Enrichment plot: OXIDATIVE_PHOSPHORYLATION
NES = -1.85
p < 0.001
Cellular Respiration
Enrichment plot: CELLULAR_RESPIRATION
NES = -1.64
p < 0.001
Aerobic Respiration
Enrichment plot: AEROBIC_RESPIRATION
NES = -1.74
p < 0.001
Control
MitolΔ/Δ
Enrichment score (ES)
Ranked list metric (Signal2Noise)
Rank in Ordered Dataset
Zero cross at 4272
Enrichment profile — Hits Ranking metric scores
B
Unfolded Protein Response
Enrichment plot: HALLMARK_UNFOLDED_PROTEIN_RESPONSE
NES = -1.56
p < 0.001
Response to ER stress
Enrichment plot: GO_RESPONSE_TO_ER_STRESS
NES = -1.21
p < 0.05
IRE1 mediated unfolded protein response
Enrichment plot: GOBP_IRE1_MEDIATED_UNFOLDED_PROTEIN_RESPONSE
NES = -1.309
p < 0.05
Response to topologically incorrect proteins
Enrichment plot: RESPONSE_TO_TOPOLOGICALLY_INCORRECT_PROTEINS
NES = -1.34
p < 0.01
C
Ddit3
Gene Set Score
ns
Control
MitolΔ/Δ
D
Dnm1l
Mfn1
Mfn2
ns
E
Atad3a
Pink1
Prkn
Atg5
Atg7
Ulk1
Bnip3l
*
F
Marchf5 (Mitol)
Bax
Bak1
Relative mRNA expression
****
*
Control
MitolΔ/Δ

Figure EV3. Mitol involvement in mitochondrial function and mitophagy.

(**A**, **B**) GSEA analysis of metabolic genes involved in ER and mitochondrial activity. (**C**) Gene encoding CHOP ($n = 4$). (**D**) Genes encoding DRP1, MFN1 and MFN2, respectively, which are involved in mitochondrial fusion and fission ($n = 4$). (**E**) Genes which are involved in Pink-Parkin mediated mitophagy pathway ($n = 3$). (**F**) RT-qPCR analysis showing genes expression levels in murine LT-HSC after 1 week of Poly:IC injection ($n = 5$). Data Information: In EV3A, B, P value of the normalized enrichment score (NES) was analyzed with an empirical phenotype-based permutation test. In EV3C-E, the box plot's central band marks the median, boxes mark the first and third quartiles, and whiskers extend the boxes to the largest value no further than 1.5 times the interquartile range. Gene Set Score was analyzed with the Wald test. In EV3F, data represent mean ± SEM with two-tailed unpaired Student's t test. ns, $P > 0.05$; *$P < 0.05$; ****$P < 0.0001$.

© The Author(s)

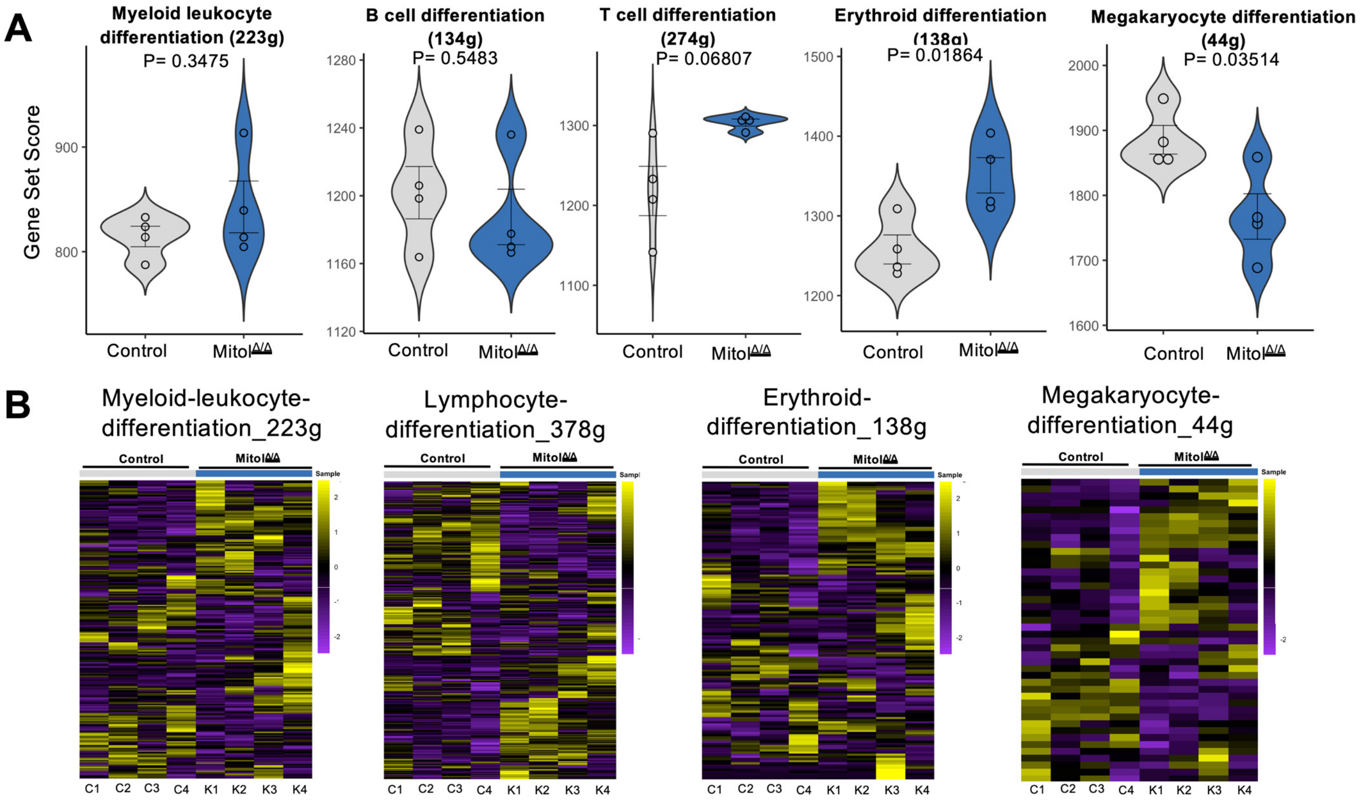

Figure EV4. Distribution of lineage programming of Mitol$^{Δ/Δ}$ HSCs shown in RNA-seq analysis.

(**A**, **B**) Distribution of lineage programs in Mitol$^{Δ/Δ}$ HSCs 1 week post Poly:IC induction was shown in (**A**) Violin plots and heatmaps (**B**) by RNA-seq analysis ($n = 4$). Data Information: In EV4A, horizontal lines in violin plots represent quartiles. Data was analyzed with the Wald test.

© The Author(s)

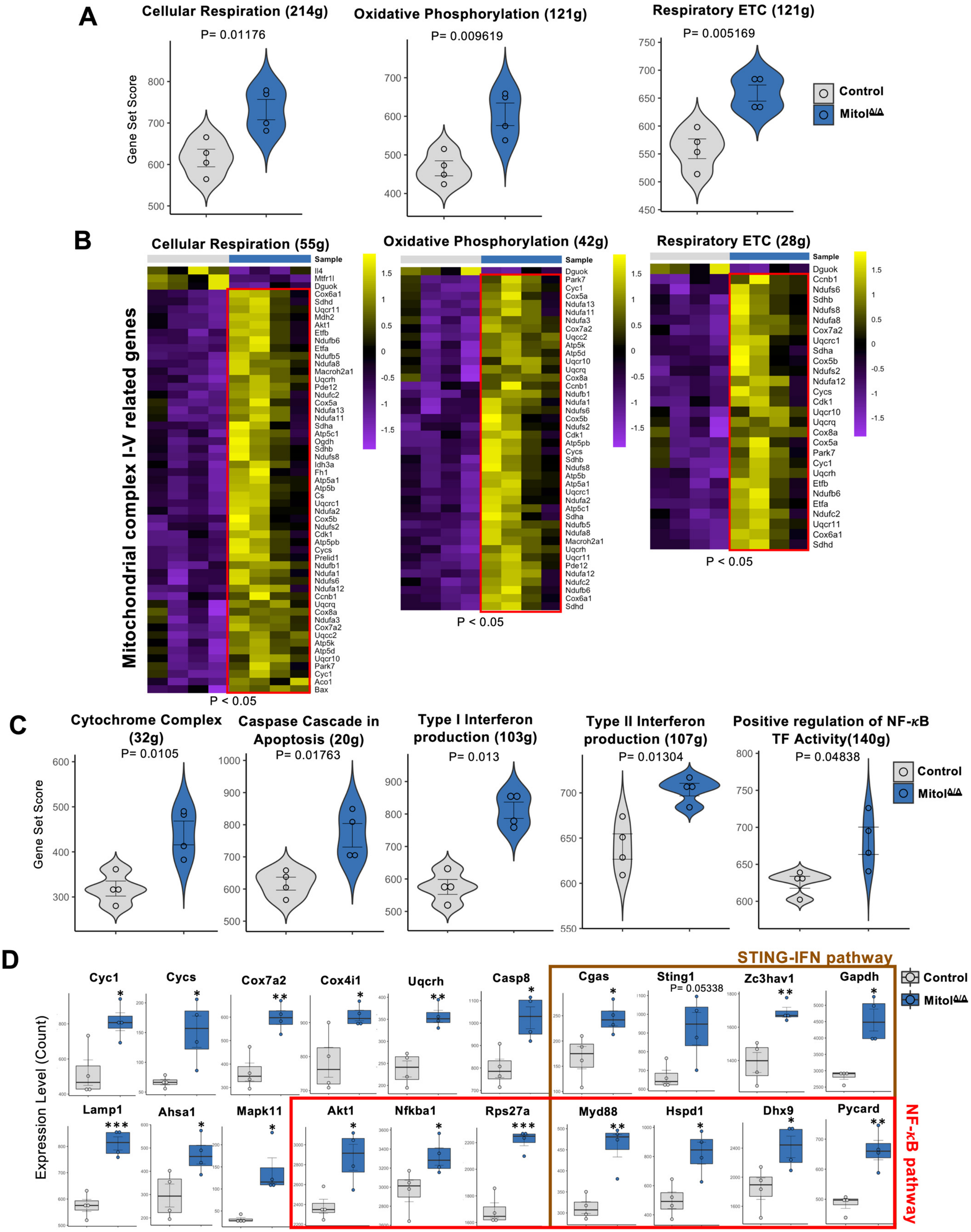
A
Cellular Respiration (214g)
P= 0.01176
Oxidative Phosphorylation (121g)
P= 0.009619
Respiratory ETC (121g)
P= 0.005169
Gene Set Score
Control
MitolΔ/Δ
B
Cellular Respiration (55g)
Oxidative Phosphorylation (42g)
Respiratory ETC (28g)
Sample
Mitochondrial complex I-V related genes
P < 0.05
C
Cytochrome Complex (32g)
P= 0.0105
Caspase Cascade in Apoptosis (20g)
P= 0.01763
Type I Interferon production (103g)
P= 0.013
Type II Interferon production (107g)
P= 0.01304
Positive regulation of NF-κB TF Activity(140g)
P= 0.04838
Gene Set Score
Control
MitolΔ/Δ
D
STING-IFN pathway
Cyc1
Cycs
Cox7a2
Cox4i1
Uqcrh
Casp8
Cgas
Sting1
P= 0.05338
Zc3hav1
Gapdh
Lamp1
Ahsa1
Mapk11
Akt1
Nfkba1
Rps27a
Myd88
Hspd1
Dhx9
Pycard
NF-κB pathway
Expression Level (Count)
Control
MitolΔ/Δ

 © The Author(s)

Figure EV5. Enhanced mitochondrial respiration by Mitol regulates apoptotic pathways.

(**A**) Violin plots showed enhanced mitochondrial respiration in Mitol$^{\Delta/\Delta}$ HSCs ($n = 4$). (**B**) Heatmaps showed significantly upregulated genes in mitochondrial respiration caused by Mitol depletion. Red box highlights genes encoding mitochondria complex I–V. (**C**) Violin plots showed activated pathways associated with enhanced mitochondrial metabolism in Mitol$^{\Delta/\Delta}$ HSCs ($n = 4$). (**D**) Box plots showed significantly upregulated genes involved in pathways shown in violin plots (**C**) ($n = 4$). Borrow color highlights genes involved in STING-IFN pathway and red color highlights genes involved in NF-κB pathway. Data Information: In EV5A,C, horizontal lines in violin plots represent quartiles. In EV5D, the box plot's central band marks the median, boxes mark the first and third quartiles, and whiskers extend the boxes to the largest value no further than 1.5 times the interquartile range. Data was analyzed with the Wald test. ns, $P > 0.05$; $^{*}P < 0.05$; $^{**}P < 0.01$; $^{***}P < 0.001$.

© The Author(s)
